# Exploring the predation of large land snails using preyed shell remains from rock anvil sites in a tropical limestone rainforest in Malaysia

**DOI:** 10.3897/BDJ.10.e90063

**Published:** 2022-09-30

**Authors:** Siew-Yin Woo, Junn Kitt Foon, Thor-Seng Liew

**Affiliations:** 1 Institute for Tropical Biology and Conservation, Universiti Malaysia Sabah, Kota Kinabalu, Malaysia Institute for Tropical Biology and Conservation, Universiti Malaysia Sabah Kota Kinabalu Malaysia; 2 Australian Museum Research Institute, Australian Museum, Sydney, Australia Australian Museum Research Institute, Australian Museum Sydney Australia

**Keywords:** karst, Cyclophoridae, Camaenidae, Ariophantidae, Muscicapidae

## Abstract

The study of prey-predator interactions between land snails and birds offers important insights into evolutionary and ecological relationships. Here, we report a case study of rock anvils presumably used by the birds *Myophonuscaeruleus* and *Enicurusruficapillus* in a cave cavity of a limestone hill in Malaysia. We did not detect any other species in the plots and, therefore, based on our short study duration, we cannot rule out the possibility that other species, such as mammals, preyed on the snails. The predated shell remains of four land snails namely, *Hemiplecta* sp., *Cyclophorusperdixperdix*, *Amphidromusatricallosusperakensis* and *Cyclophorussemisulcatus*, were found around rock anvils in the nine plots. Finally, we discussed the potential and the limitations of using shell remains of preyed land snails for behavioural, ecological and evolutionary studies between land snails and their predators.

## Introduction

Land snails play a significant role in the terrestrial ecosystem as an important food source for many organisms, including birds and mammals (Barker 2004, Bentley-Condit and Smith 2010, Rosin et al. 2011, Morii et al. 2021, Hashim et al. 2021). However, very little is known about these ecological interactions in tropical terrestrial ecosystems of Southeast Asia, as there are very few systematic in-situ studies on land snail predation in the region and almost none for macro land snails of shell sizes larger than 10 mm (Bennett 2014, Liew and Schilthuizen 2014). Elsewhere, only a few in-situ studies of tropical land snail predation by vertebrates have been undertaken (Kasigwa et al. 1983, Efe et al. 2015, Kraemer et al. 2019). There are a few reasons why these field studies are rare for macro land snails in tropical terrestrial ecosystems in Southeast Asia. First, the density of large-sized land snails in tropical rainforests is generally low, except in forests on calcareous habitats, such as limestone karst ecosystems (Schilthuizen et al. 2003, Liew et al. 2008). Second, the shelled remains of the preyed snails could only persist for a very short time in the acidic conditions of non-limestone forests compared to limestone forests (Pearce 2008, Říhová et al. 2018, Němec and Horsák 2019). Finally, direct observation or field experiment on the interaction of land snails and their predators is difficult given the structural complexity of forest stands and herbaceous vegetation and the diversity of predators in tropical regions, apart from chance observations (e.g. Ihlow et al. 2012, Lima et al. 2017).

Interactions between land snails and birds offer important insights into evolutionary and ecological relationships (Vermeij 1982, Vermeij 1993, Vermeij 1995, Graveland 1996, Graveland and van der Wal 1996, Mänd et al. 2000, Hoso 2012, Morii et al. 2016, Bańbura et al. 2020). Birds are the best known and most observed shell-breaking predators of land snails (Vermeij 2015). Selective predation by birds is one of the main mechanisms responsible for variation in shell polymorphism (Rosin et al. 2011, Kraemer et al. 2019). They remove, break or crush the shells with teeth, claws or beaks (Norris and Johnstone 1988, Rosin et al. 2011, Helwerda and Schilthuizen 2014, Kraemer et al. 2019, Johnson 2021).

**Figure 1.**
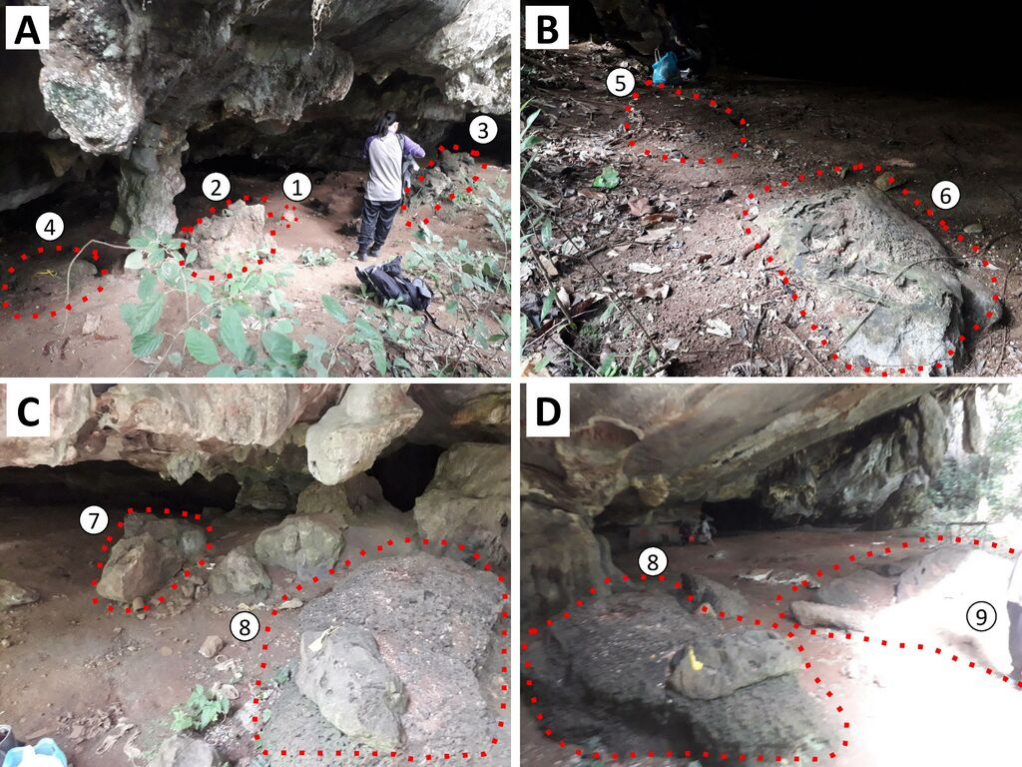
Sampling plots in Phg 77 Bukit Mengapur, Pahang, Malaysia (3°44'47.0" N, 102°50'17.9" E). (A) Plots K1, K2, K3 and K4; (b) Plots K5 and K6; (C) Plots K7 and K8; (D) Plots K8 and K9.

Although birds can swallow smaller land snails whole, when eating larger snails, birds often carry the snail to the nearest solid object such as rocks, empty bottles or stumps of felled trees to break its shell (Morris 1954, Goodhart 1958, Richardson 1975, Rosin et al. 2011, Wada et al. 2012, Shikov and Vinogradov 2013, Efe et al. 2015, Kraemer et al. 2019, Kwieciński et al. 2019). However, only two of these observations come from the tropical region (Efe et al. 2015, Kraemer et al. 2019). Therefore, rock anvils in limestone habitats are an ideal natural laboratory for studying large land snails and specific predators, especially birds, due to the high number of living snail populations, good preservation of preyed shells and availability of rock anvils in the open cave next to the forest (Fig. 1).

It is known that the marks left by predators on the shells of marine and freshwater molluscs include injuries and scars at the aperture (Ebbestad et al. 2009, Kröger 2011), on the shell whorls (Dietl and Hendricks 2006, Stafford et al. 2015, Sime and Kelley 2016, Harper 2016, Tluste et al. 2020) and as boreholes (Kowalewski 2004, Sime and Kelley 2016, Harper 2016). On the other hand, traces of predation on land snail shells include boreholes (Liew and Schilthuizen 2014), remains of diptera pupae on the inside of the shell (Tluste et al. 2020) and breakage on the aperture and shell whorls (Němec and Horsák 2019, Hayashi and Sugiura 2021).

Given the potential for documenting predation in the natural laboratory of a tropical limestone habitat, we report on a case study of rock anvils presumably used by birds in a cave cavity of a limestone hill in Malaysia. The main objective of this study is to document the preyed snails at selected rock anvils and to investigate the temporal patterns of accumulation of preyed shells at these sites.

## Materials and Methods

A total of nine plots were established around the selected rock anvils of different sizes along the cave of Phg 77 Bukit Mengapur, Pahang, Malaysia (3°44'47.0" N, 102°50'17.9" E) (Fig. 1; Liew et al. 2021). In the field, we identified rock anvil sites as a plot where the preyed shells were found on or next to rocks. The rock anvils in each of the nine plots varied in size and shape. Each of the plots with preyed shells and rock anvils is considered independent of each other, as the distance between the rock anvils and the preyed shells left by the predators can be clearly determined for each rock anvil.

In each plot, we collected all shells larger than 15 mm in width or height within 50 cm of the rock anvils because we assumed that snails smaller than 15 mm might have been completely swallowed by birds or that the fragments were too small for meaningful analysis. Each shell was then carefully examined to determine whether it was an individual shell, which usually had the aperture and shell columella fully or partially intact, or a shell fragment, where it was not possible to determine whether or not it was a fragment from one or another individual from the same plot. All plots were sampled twice. The first sampling took place on 13 January 2019 and all shells were collected. The second sampling was carried out on 7 March 2020.

To detect and confirm the presence of the species that frequented the rock anvil plots, we set up a total of 10 camera traps (model: Reconyx HyperFire HC500 Semi - Covert IR) for 3 days (30 trap days in total), three on plot K7 and seven on plots K8 and K9 during the second sampling. The cameras were set at a height of 30–40 cm above the ground, as the target animals were small and medium-sized animals and the field of view of the cameras covered the rock anvil in the plot. The camera traps were in operation day and night. As there is always poor light in the caves, the camera traps use an infrared flash that produces black and white photos.

Afterwards, all the shells were cleaned and then oven dried. The specimens were deposited in the BORNEENSIS collection of Universiti Malaysia Sabah, under reference numbers: BORMOL 14623, 14625, 14627–14628, 14630–14633, 14635–14636, 14650–14667; 14669–14674; 14676–14677, 14955–14968, 14970–14972, 14974–14996. For species identification, the morphologically based identifications were done at species level, based on the checklist of limestone karst dwelling land snails in Perak published by Foon et al. (2017). The number of snail shells was tabulated according to the plots, year of sampling and land snail species. Any tiny shell fragments that could not be identified were excluded from the dataset.

## Results

**Figure 2.**
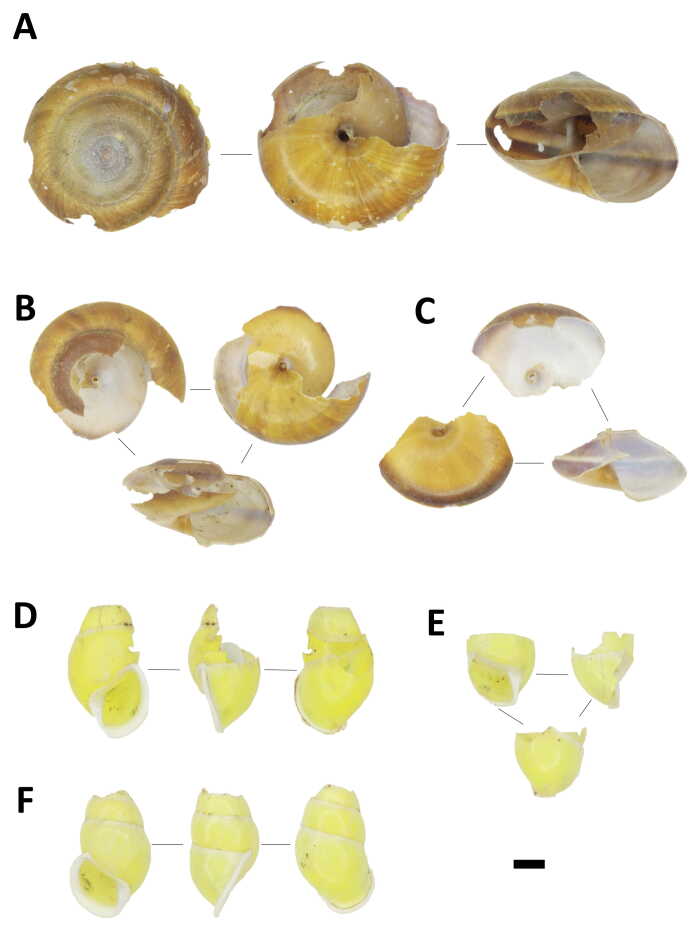
Shells damaged by predators, collected from rock anvils. (A)–(C) *Hemiplecta* sp. (Ariophantidae), BORMOL 14979; (D)–(F) *Amphidromusatricallosusperakensis* (Camaenidae), BORMOL 14996. Scale = 1 cm.

**Figure 3.**
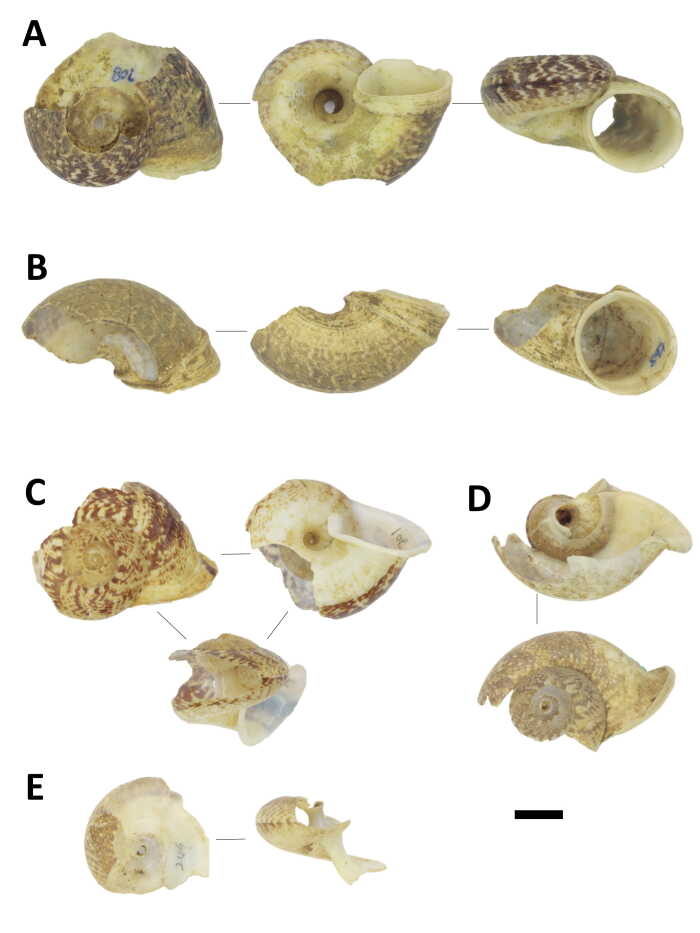
Shells damaged by predators, collected from rock anvils. (A)–(B) *Cyclophorussemisulcatus* (Cyclophoridae), BORMOL 14807; (C)–(E) *Cyclophorusperdixperdix* (Cyclophoridae), BORMOL 14978. Scale = 1 cm.

**Figure 4.**
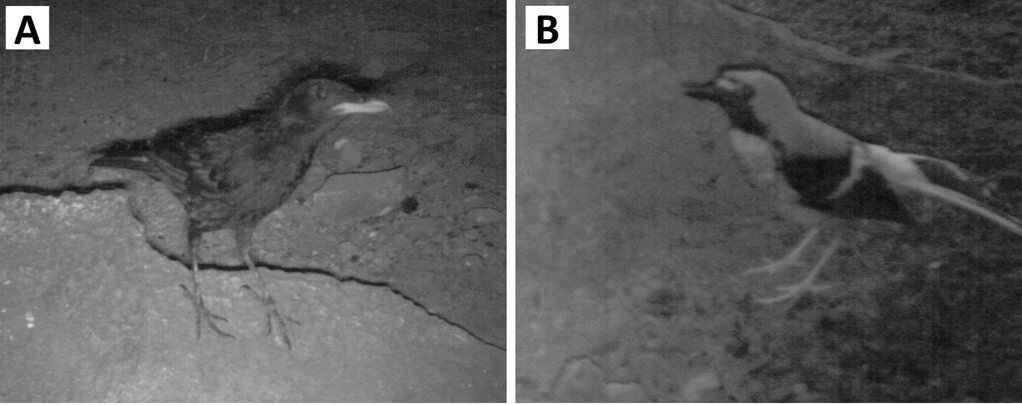
Two bird species were recorded with the camera traps set up on plots 7, 8 and 9 in Phg 77 Bukit Mengapur, Pahang, Malaysia (3°44'47.0" N, 102°50'17.9" E). (A) Blue-whistling Thrush, *Myophonuscaeruleus*, recorded with the camera trap on 04/03/2020 at 6.22 pm; (B) Red Chestnut-naped forktail, *Enicurusruficapillus*, recorded with the camera trap on 06/03/2020 at 8.16 am.

A total of 943 shells belonging to four large-sized snail species, namely *Amphidromusatricallosusperakensis* (Camaenidae), *Hemiplecta* sp. (Ariophantidae), *Cyclophorusperdixperdix* (Cyclophoridae) and *Cyclophorussemisulcatus* (Cyclophoridae) were collected from the nine plots (Figs 2, 3; Suppl. material 1). Shell fragments larger than 1.5 cm can be distinguished as individual shells in both *Cyclophorus* species, while only 59% of *Amphidromusatricallosusperakensis* and 45% of *Hemiplecta* sp. could be confirmed as unique individuals. Therefore, differences in the number of snails (i.e. abundance) between two samplings on plots of the two species should be considered as maximum estimates and treated with caution, but the trend patterns between samplings and between plots were fairly consistent. Each of the two bird species were recorded once by camera traps installed on plots K7, K8 and K9 as potential predators of the sites, namely the Blue-whistling Thrush *Myophonuscaeruleus* on 04/03/2020 at 6.22 pm and the Red chestnut-naped forktail *Enicurusruficapillus* on 06/03/2020 at 8.16 am, both of which are from the family Muscicapidae (Fig. 4).

**Figure 5.**
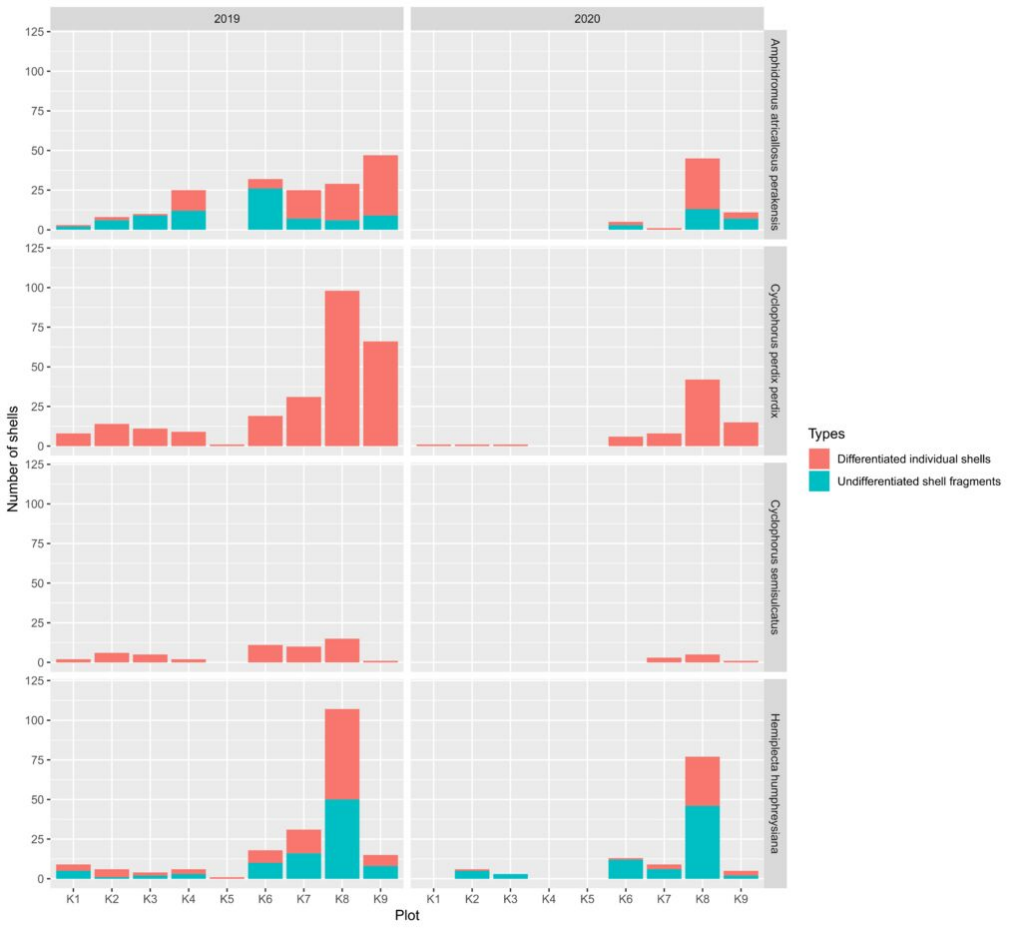
The number of shells of the four land snail species collected from the nine plots of rock anvils in Phg 77 Bukit Mengapur, Pahang, in 2019 and 2020 (3°44'47.0" N, 102°50'17.9" E). The shells collected in 2019 were accumulated at the plots before the first sampling on 13/01/2019, while the shells collected in 2020 represent the shells brought to the plots by predators between 14/01/2019 and 07/03/2020. The shell remains were either differentiated individual shells or undifferentiated shell fragments.

In 2020, a total of 258 shells were collected, brought, preyed and left by predators over a period of 418 days, between 14 January 2019 and 7 March 2020 (Fig. 5). About two-thirds of the shells (169 shells) (65%) were collected in plot K8, while about 30% of the shells were found in plot K6 (24 shells), plot K7 (21 shells) and plot K9 (32 shells). Twelve shells were found in the remaining five plots. The land snail species composition consists of 113 *Hemiplecta* sp. shells (44%), 74 *Cyclophorusperdixperdix* shells (28%), 62 *Amphidromusatricallosusperakensis* shells (24%) and nine *Cyclophorussemisulcatus* (4%).

The duration of the 685 shells that had accumulated at the sites was unknown prior to the first sampling on 13 January 2019 (Fig. 5). Nevertheless, most shells were collected in the same plots where most shells were collected in 2020 - 249 shells in plot K8 (36%), 129 shells in plot K9 (19%), 97 shells in plot K7 (14%) and 80 shells in plot K6 (12%). Similar to the shells collected in 2020, almost all shells came from the three species, *Hemiplecta* sp. (197 shells, 29%), *Cyclophorusperdixperdi*x (257 shells, 38%), *Amphidromusatricallosusperakensis* (179 shells, 26%) and C*yclophorus semisulcatus* (52 shells, 7%).

## Discussion

Our preliminary results indicate that rock anvils are a potential natural laboratory providing shell remains of land snails in the predation process, which can potentially be used for behavioural, ecological and evolutionary studies between land snails and their predators (Vermeij 1982, Vermeij 1995, Alexander and Dietl 2003). Although in our study only two bird species, *Myophonuscaeruleus* and *Enicurusruficapillus*, were recorded only once each during limited sampling at the rock anvil sites (without recording predation actions), we consider that the birds are likely the main predators of the site as the same birds were observed preying on snails at different sites (Suppl. materials 2, 3; Delacour 1942, McClure et al. 1967, Khoo 2012).

We did not detect other potential predator species in the plots, but it does not mean other species were absent due to our short duration of detection. Our camera captured small birds. We think that if the rodents occur in the place where the cameras were operating, their images should have been captured as the cameras captured the images of the small birds. This study only examined the preyed snails that were brought by the predators to rock anvils to break the shell. We cannot exclude that the same predators could also prey on other smaller snail species and juveniles of *Cyclophorus* or other larger species by swallowing the snail whole (e.g. Gonzalez-Solis et al. 1996, Allen 2004).

Birds, as well as other predators, leave some traces on the shell remains if they cannot swallow the entire snail along with its shell (Němec and Horsák 2019). Examination of the marks on the preyed shells can provide information about the different predators (Němec and Horsák 2019, but see Calderwood and Sigwart 2016), the different predation intensity or selection pressure at different sites (Dietl and Alexander 2009, Ebbestad et al. 2009, Stafford et al. 2015). For example, from comparative studies between species with different shell morphologies from the same habitat, presumably under predation pressure from the same predator, we can infer that the morphology with less predation is a better defence strategy (e.g. Shachak et al. 1981, Lindström and Peel 2010).

The preyed snails were brought by the predator to break the shells with the help of stone anvils, as the bare ground in the cave cavity is not the right habitat for the snail species documented here. Birds are known to carry the snail to a stone anvil and then smash the shell by swinging it forcefully against a rock anvil (Morris 1954, Parisi and Gandolfi 1974, Henty 1986, Khoo 2012). For most mammals, including rodents, that prey on snails, the snails were collected in their original habitat in the forest or water body, where the snails were preyed upon by the predators at the site (Parisi and Gandolfi 1974, Morii and Wakabayashi 2017, Saeki et al. 2017, Němec and Horsák 2019). However, rodents are also known to bring snails to feeding grounds, where they crack and eat them in a relatively safe habitat, such as under bushes or rocks. Shelters are crucial for rodents and rodents do not seem to prey heavily on snails in areas more than 15–20 m from a shelter (Abramsky et al. 1990, Moreno-Rueda 2008).

As shown in this study, four land snail species present at the site were selected for food by predators, presumably birds and the shell forms of the three land snail genera were different. It is not difficult to imagine that, with more predatory actions of the predator recorded on video in the future, the predatory behaviour may turn out to be very specialised when different predators (e.g. bird species) are dealing with the same land snail species or when the same predator species is dealing with different land snail species with different shell forms (e.g. Morris 1954). Our preliminary results also showed that the shells of some species break into smaller pieces more easily than those of other species. This means that it is not possible to establish beyond doubt that the smaller shell remains come from the same or different individual.

In addition, resampling and longitudinal studies are important because the overall dynamics of these two predator-prey systems varied and they also changed differently during the period studied (Cameron 1969, Mondal et al. 2014). Although we cannot confirm how long shells had been accumulating at sites in the samples collected in 2019, we can still make some interesting observations when comparing these historical records before 2019 and the recent records for just over 1 year. For example, the number of land snail species selected by the predators did not change significantly compared to the previous records. Similarly, predators used the same rock anvils more frequently than other adjacent rock anvils, based on historical and recent records.

This non-invasive method for studying predation could have a lot of potential, but it also has its limitations (Tluste et al. 2020). First, although the analysis of empty preyed shells is a powerful method, predators that eat the whole snail could be a problem for assessing selection by predators (Němec and Horsák 2019). At the same time, the density of preyed shells could be used as a predictor of predation frequency and density by predators on land snails (Thurman et al. 2008, Stafford and Leighton 2011, Říhová et al. 2018), although the estimate could be biased without simultaneous surveys of live populations of land snails and predators, as well as proper documentation of predatory events (Stafford and Leighton 2011, Dietl and Kosloski 2013). Finally, not all prey species can be studied using this method, as the shells of some land snail species may be broken into pieces that are too difficult to discern whether they came from the same or a different individual.

Therefore, this study needs further direct observational data to support the indirect evidence of predator-snail interactions at this site so that these data can be uploaded and disseminated through Global Biotic Interactions (GloBI), globalbioticinteractions.org (Poelen et al. 2014, Jordano 2021). Whenever possible, it is useful to supplement the preyed shell data with in-situ experiments (e.g. Morii and Wakabayashi 2017) to document some of the predator-prey interactions in the wild that may not be known.

## Supplementary Material

02768F9F-2795-5861-ABBE-97ADA3CA2CC4 10.3897/BDJ.10.e90063.suppl1 Supplementary material 1 A dataset of the specimens tabulated by plot included in this study. Data type Data Table Brief description The dataset contains a tabulation-delimited table with 22 fields in Darwin Core terms and 76 records containing sampling, taxonomic and collection information. File: oo_711784.txt https://binary.pensoft.net/file/711784 Siew-Yin Woo, Junn-Kitt Foon, Thor-Seng Liew

B48DBEF4-4946-5FD1-A0D6-73F78DCF595C 10.3897/BDJ.10.e90063.suppl2 Supplementary material 2 The blue whistling thrush, *Myophonuscaeruleus*, photographed with camera trap at a rock anvil of Gunung Kanthan. Data type Photo Brief description The blue whistling thrush, *Myophonuscaeruleus*, photographed with camera trap at a rock anvil of Gunung Kanthan (N 4.76293, 101.12007). (A) Recorded on 19/01/2019 at 1:04 pm; (B) Recorded on 24/02/2019 at 10:36 am; (C) Recorded on 29/01/2019 at 1:07 pm. File: oo_664298.docx https://binary.pensoft.net/file/664298 Siew-Yin Woo, Junn-Kitt Foon, Thor-Seng Liew

A1161E74-60F7-5F43-9542-761A07D1C12E 10.3897/BDJ.10.e90063.suppl3 Supplementary material 3 The blue whistling thrush, *Myophonuscaeruleus*, was captured on camera smashing the freshwater snail *Pomacea* sp. on the rock anvil at Bukit Jernih. Data type Photo and video Brief description The blue whistling thrush, *Myophonuscaeruleus*, was captured on camera smashing the freshwater snail *Pomacea* sp. on the rock anvil at Bukit Jernih Recreation Park (6° 32' 46.83" N, 100° 16' 9.15" E) near the limestone hill Prs 25 Bukit Jerneh in Perlis on 26/05/2016 at 2:56 pm. File: oo_664300.docx https://binary.pensoft.net/file/664300 Siew-Yin Woo, Junn-Kitt Foon, Thor-Seng Liew
