# Supplementary material for: Exploring the predation of large land snails using preyed shell remains from rock anvil sites in a tropical limestone rainforest in Malaysia
Source: Biodivers Data J. 2022 Sep 30;10:e90063. doi: 10.3897/BDJ.10.e90063 (PMC9836610; doi:10.3897/BDJ.10.e90063)
Supplement: Supplementary material 2 — The blue whistling thrush, Myophonuscaeruleus, photographed with camera trap at a rock anvil of Gunung Kanthan. [file bdj-10-e90063-s002.docx]

**Suppl. material 1:**

**Authors:** Siew-Yin Woo, Junn-Kitt Foon, Thor-Seng Liew

**Data type:** Photo.

**Brief description:**

The blue whistling thrush, *Myophonus caeruleus*, photographed with camera trap at a rock anvil of Gunung Kanthan (N 4.76293, 101.12007). (A) Recorded on 19/01/2019 at 1:04 pm; (B) Recorded on 24/02/2019 at 10:36 am; (C) Recorded on 29/01/2019 at 1:07 pm.

**
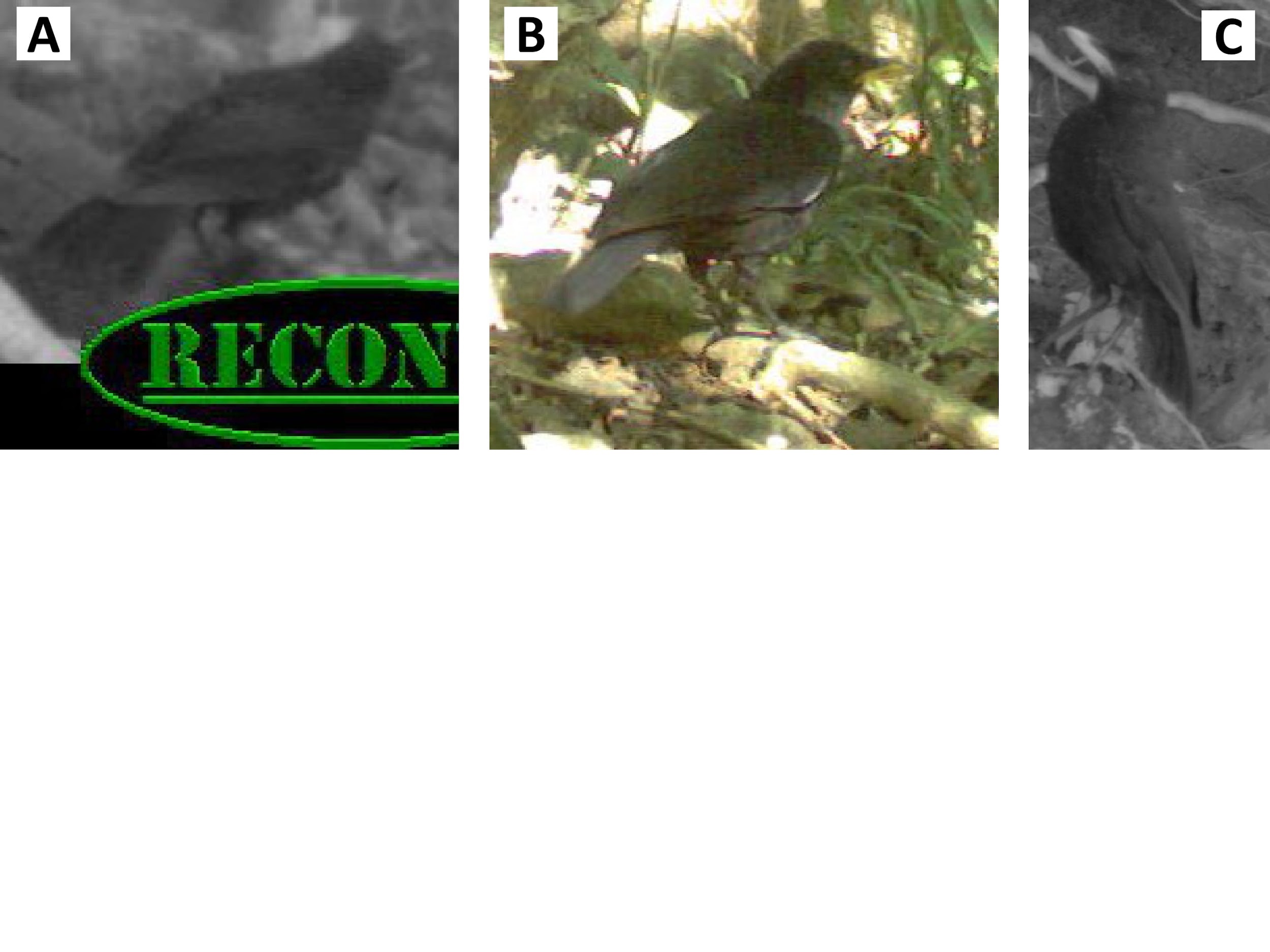
**
