# Supplementary material for: Exploring the predation of large land snails using preyed shell remains from rock anvil sites in a tropical limestone rainforest in Malaysia
Source: Biodivers Data J. 2022 Sep 30;10:e90063. doi: 10.3897/BDJ.10.e90063 (PMC9836610; doi:10.3897/BDJ.10.e90063)
Supplement: Supplementary material 3 — The blue whistling thrush, Myophonuscaeruleus, was captured on camera smashing the freshwater snail Pomacea sp. on the rock anvil at Bukit Jernih. [file bdj-10-e90063-s003.docx]

**Suppl. material 2:**

**Authors:** Siew-Yin Woo, Junn-Kitt Foon, Thor-Seng Liew

**Data type:** Video and photo.

**Brief description:**

The blue whistling thrush, *Myophonus caeruleus*, was captured on camera smashing the freshwater snail *Pomacea* sp. on the rock anvil at Bukit Jernih Recreation Park (6° 32' 46.83" N, 100° 16' 9.15" E) near the limestone hill Prs 25 Bukit Jerneh in Perlis on 26/05/2016 at 2:56 pm.

**
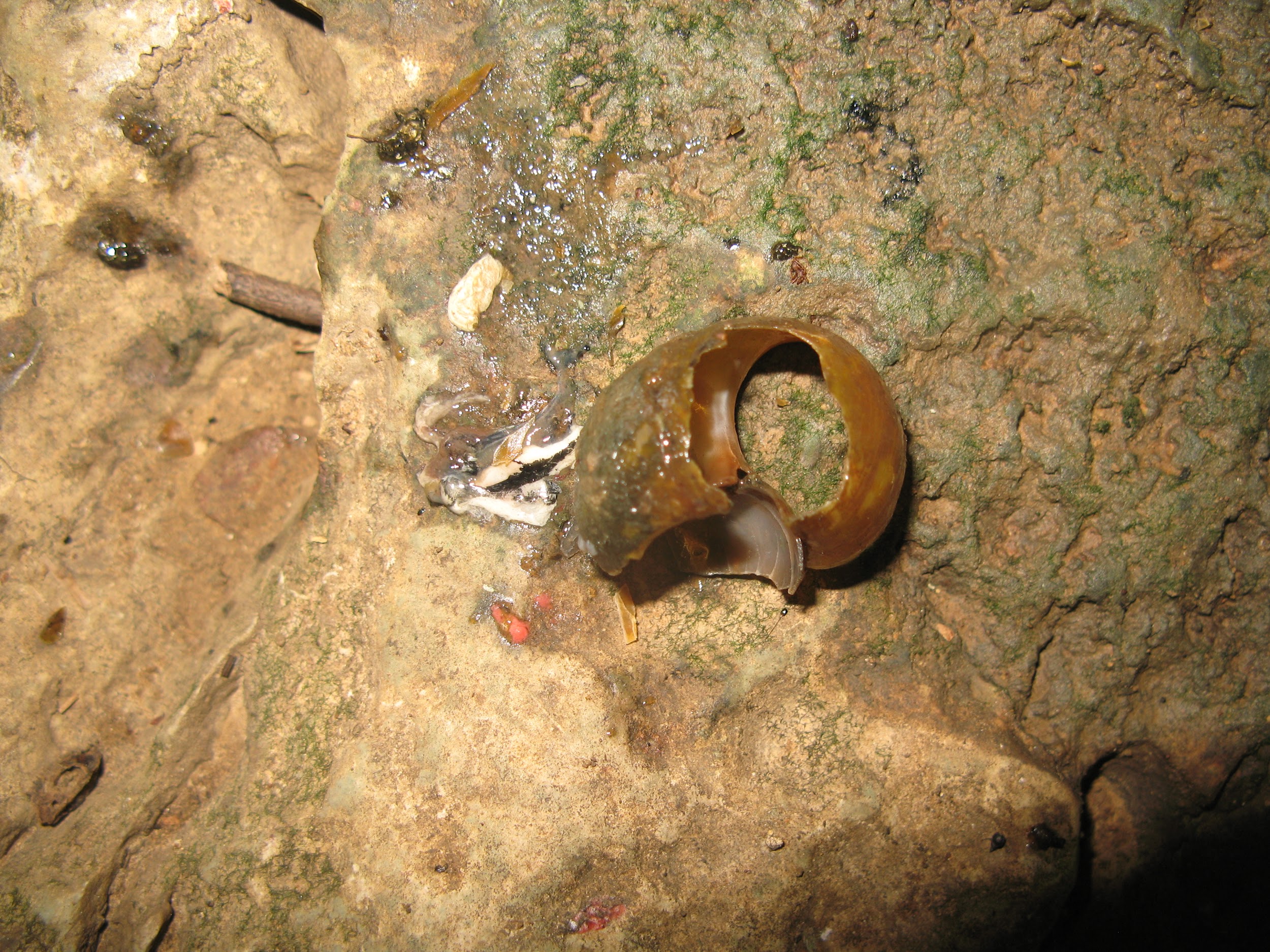
**
